# (*E*)-6-Amino-1,3-dimethyl-5-[(pyridin-2-yl­methyl­idene)amino]­pyrimidine-2,4(1*H*,3*H*)-dione

**DOI:** 10.1107/S1600536811031618

**Published:** 2011-08-11

**Authors:** Irvin Booysen, Thulani Hlela, Muhammed Ismail, Thomas Gerber, Eric Hosten, Richard Betz

**Affiliations:** a University of Kwazulu-Natal, School of Chemistry, Private Bag X01, Scottsville 3209, Pietermaritzburg, South Africa; b Nelson Mandela Metropolitan University, Summerstrand Campus, Department of Chemistry, University Way, Summerstrand, PO Box 77000, Port Elizabeth 6031, South Africa

## Abstract

In the title compound, C_12_H_13_N_5_O_2_, a Schiff-base-derived chelate ligand, the non-aromatic heterocycle and its substituents essentially occupy one common plane (r.m.s. of fitted non-H atoms = 0.0503 Å). The N=C bond is *E*-configured. Intra­cyclic angles in the pyridine moiety cover the range 117.6 (2)–124.1 (2)°. Intra- and inter­molecular N—H⋯N and N—H⋯O hydrogen bonds are observed in the crystal structure, as are intra- and inter­molecular C—H⋯O contacts which, in total, connect the mol­ecules into a three-dimensional network. The shortest ring-centroid-to-ring-centroid distance of 3.5831 (14) Å is between the two different types of six-membered rings.

## Related literature

For the crystal structures of two polymorphs of 6-amino-1,3-dimethyl-5-[(*E*-2-(methyl­sulfan­yl)benzyl­idene­amino]­pyrim­idine-2,4(1*H*,3*H*)-dione, see: Booysen *et al.* (2011*a* ▶,*b* ▶). For graph-set analysis of hydrogen bonds, see: Etter *et al.* (1990 ▶); Bernstein *et al.* (1995 ▶). For puckering analysis, see: Cremer & Pople (1975 ▶). For general information about the chelate effect in coordination chemistry, see: Gade (1998 ▶).
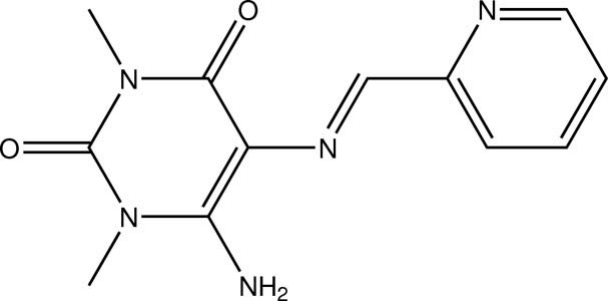


## Experimental

### 

#### Crystal data

C_12_H_13_N_5_O_2_
*M* *_r_* = 259.27
Orthorhombic, 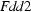

*a* = 26.5036 (8) Å
*b* = 28.9987 (14) Å
*c* = 6.2193 (1) Å
*V* = 4780.0 (3) Å^3^
*Z* = 16
Mo *K*α radiation
μ = 0.10 mm^−1^
*T* = 200 K
0.27 × 0.14 × 0.06 mm

#### Data collection

Bruker APEXII CCD diffractometer
11316 measured reflections
1620 independent reflections
1171 reflections with *I* > 2σ(*I*)
*R*_int_ = 0.049

#### Refinement

*R*[*F* ^2^ > 2σ(*F* ^2^)] = 0.039
*wR*(*F* ^2^) = 0.079
*S* = 0.94
1620 reflections
182 parameters
1 restraint
H atoms treated by a mixture of independent and constrained refinement
Δρ_max_ = 0.17 e Å^−3^
Δρ_min_ = −0.19 e Å^−3^

### 

Data collection: *APEX2* (Bruker, 2010 ▶); cell refinement: *SAINT* (Bruker, 2010 ▶); data reduction: *SAINT*; program(s) used to solve structure: *SHELXS97* (Sheldrick, 2008 ▶); program(s) used to refine structure: *SHELXL97* (Sheldrick, 2008 ▶); molecular graphics: *ORTEP-3* (Farrugia, 1997 ▶) and *Mercury* (Macrae *et al.*, 2008 ▶); software used to prepare material for publication: *SHELXL97* and *PLATON* (Spek, 2009 ▶).

## Supplementary Material

Crystal structure: contains datablock(s) I, global. DOI: 10.1107/S1600536811031618/lh5299sup1.cif

Supplementary material file. DOI: 10.1107/S1600536811031618/lh5299Isup2.cdx

Structure factors: contains datablock(s) I. DOI: 10.1107/S1600536811031618/lh5299Isup3.hkl

Supplementary material file. DOI: 10.1107/S1600536811031618/lh5299Isup4.cml

Additional supplementary materials:  crystallographic information; 3D view; checkCIF report

## Figures and Tables

**Table 1:** Hydrogen-bond geometry (Å, °)

*D*—H⋯*A*	*D*—H	H⋯*A*	*D*⋯*A*	*D*—H⋯*A*
N4—H71⋯N5^i^	0.93 (3)	2.08 (3)	2.928 (3)	151 (3)
N4—H72⋯N3	0.84 (2)	2.25 (2)	2.661 (3)	110.2 (18)
N4—H72⋯O2^ii^	0.84 (2)	2.54 (2)	3.108 (3)	125.8 (19)
C7—H7⋯O2	0.95	2.17	2.847 (3)	127
C11—H11⋯O1^iii^	0.95	2.54	3.313 (3)	138

Symmetry codes: (i) 
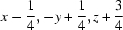
; (ii) 
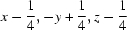
; (iii) 
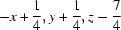
.

**Fig. 1.**
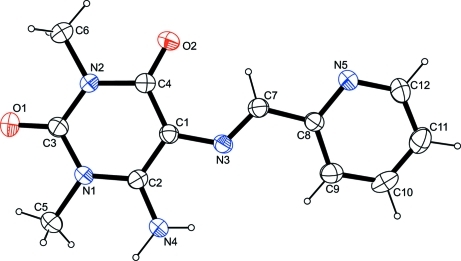
The molecular structure of the title compound, with anisotropic displacement ellipsoids (drawn at 50% probability level).

**Fig. 2.**
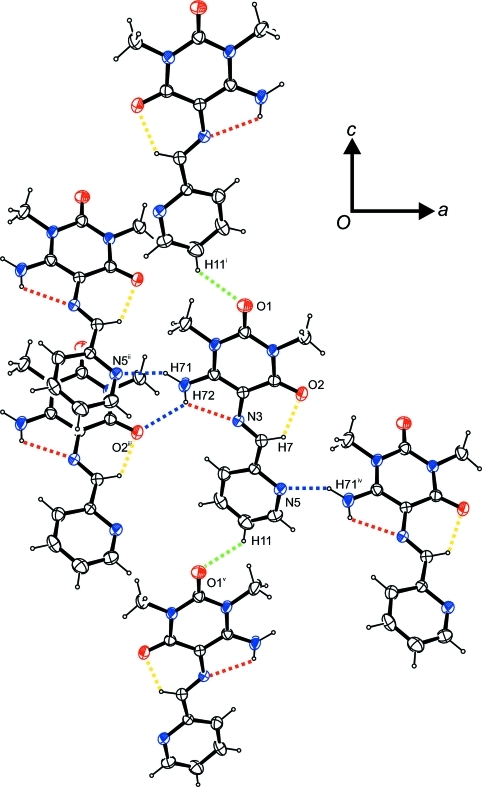
Intermolecular contacts, viewed along [0 - 1 0]. Blue dashed lines indicate clasical intermolecular hydrogen bonds, red dashed lines indicate classical intramolecular hydrogen bonds, green dashed lines intermolecular C–H···O contacts and yellow dashed lines intramolecular C–H···O contacts. Symmetry operators: i -x + 1/4, y - 1/4, z + 7/4; iix - 1/4, -y + 1/4, z + 3/4; iiix - 1/4, -y + 1/4, z - 1/4; ivx + 1/4, -y + 1/4, z - 3/4; v -x + 1/4, y + 1/4, z - 7/4.

**Fig. 3.**
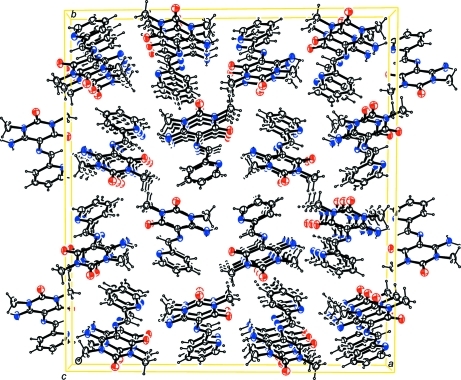
Molecular packing of the title compound, viewed along [0 0 - 1] (anisotropic displacement ellipsoids drawn at 50% probability level).

## supplementary crystallographic information

### Comment

Chelate ligands have found widespread use in coordination chemistry due to the enhanced thermodynamic stability of resultant coordination compounds in relation to coordination compounds exclusively applying comparable monodentate ligands (Gade 1998). Combining different sets of donor atoms in one chelate ligand molecule, a probe for testing and accomodating metal centers of different Lewis acidities is at hand. To enable comparative studies with envisioned coordination compounds, we determined the crystal structure of the title compound. Two crystal structures of 6-amino-1,3-dimethyl-5-[(*E*-2- (methylsulfanyl)benzylideneamino]pyrimidine-2,4(1*H*,3*H*)-dione are apparent in the literature (Booysen *et al.*, 2011*a*; Booysen *et al.*, 2011*b*).

The molecule is a Schiff-base composed of a pyridyl moiety and a 6-amino-1,3- dimethylpyrimidine-2,4(1*H*,3*H*)-dione moiety. The C=N double-bond is (*E*)-configured. A conformation analysis of the non-aromatic six-membered ring (Cremer & Pople, 1975) fails due to the low puckering amplitude (τ = 2.9 °; r.m.s. of fitted non-hydrogen atoms – including the exocyclic substituents – = 0.0503 Å). Intracyclic angles in the pyridyl moiety cover a range from 117.6 (2)–124.1 (2) ° with the smallest angle found on the nitrogen atom and the largest angle found on the unsubstituted carbon atom in *ortho* position to the nitrogen atom. The least-squares planes defined by the respective atoms of the six-membered heterocycles intersect at an angle of 8.11 (12) °. The amino group is almost planar. The plane defined by its atoms and the least-squares plane defined by the atoms of its six-membered carrier ring enclose an angle of 9.56(2.54) ° (Fig. 1).

In the crystal structure, hydrogen bonds of N–H···N type as well as N–H···O type are observed. These are intra- as well as intermolecular and, in the case of N–H···N hydrogen bonds, involve only the nitrogen atom of the aromatic system and the Schiff base's double bonded nitrogen atom as acceptor. The intramolecular hydrogen bond shows bifurcation involving an oxygen atom. Apart from these classical hydrogen bonds, intra- as well as intermolecular C–H···O contacts can be observed whose range falls by more than 0.5 Å (in the former case) and by almost 0.2 Å (in the latter case) below the sum of van-der-Waals radii of the atoms participating. While the intramolecular C–H···O contact is apparent between the vinylic hydrogen atom and the neighbouring oxygen atom, the intermolecular C–H···O contacts are supported by the C–H group in *para* position to the Schiff-base substituent on the aromatic system and the keto group's oxygen atom that is not involved in the intramolecular C–H···O contacts. In terms of graph-set analysis (Etter *et al.*, 1990; Bernstein *et al.*, 1995), the descriptor for the classical hydrogen bonds is *S*(5)*C*^1^_1_(6)*C*^1^_1_(8) on the unitary level while a description of the C–H···O contacts necessitates a *S*(6)*C*^1^_1_(12) descriptor on the same level. In total, the molecules are connected to a three-dimensional network. The shortest intercentroid distance between two centers of gravity was measured at 3.5831 (14) Å while the shortest intercentroid distance between two aromatic systems was found at 5.2956 (14) Å. (Fig. 2).

The packing of the title compound in the crystal is shown in Figure 3.

### Experimental

Equimolar amounts of picolinaldehyde (1.00 g, 9.36 mmol) and 5,6-diamino-1,3-dimethylpyrimidine-2,4(1*H*,3*H*)-dione (1.59 g) in anhydrous methanol (50 cm^3^) were refluxed for 3 h. The reaction mixture was allowed to cool to room temperature. An orange precipitate was isolated, which was recrystallized from anhydrous acetonitrile to give orange crystals. The crystals were filtered and dried under vacuum.

### Refinement

Carbon-bound H atoms were placed in calculated positions (C—H 0.95 Å) and were included in the refinement in the riding model approximation, with *U*(H) set to 1.2*U*_eq_(C). The H atoms of the methyl groups were allowed to rotate with a fixed angle around the C—C bond to best fit the experimental electron density (HFIX 137 in the *SHELX* program suite (Sheldrick, 2008)), with *U*(H) set to 1.5*U*_eq_(C). Both nitrogen-bound H atoms were located in a difference Fourier map and refined freely.

### Crystal data

**Table:**

C_12_H_13_N_5_O_2_	*F*(000) = 2176
*M**_r_* = 259.27	*D*_x_ = 1.441 Mg m^−^^3^
Orthorhombic, *F**d**d*2	Mo *K*α radiation, λ = 0.71073 Å
Hall symbol: F 2 -2d	Cell parameters from 2610 reflections
*a* = 26.5036 (8) Å	θ = 2.8–25.2°
*b* = 28.9987 (14) Å	µ = 0.10 mm^−^^1^
*c* = 6.2193 (1) Å	*T* = 200 K
*V* = 4780.0 (3) Å^3^	Platelet, red
*Z* = 16	0.27 × 0.14 × 0.06 mm

### Data collection

**Table:**

Bruker APEXII CCD diffractometer	1171 reflections with *I* > 2σ(*I*)
Radiation source: fine-focus sealed tube	*R*_int_ = 0.049
graphite	θ_max_ = 28.3°, θ_min_ = 2.1°
φ and ω scans	*h* = −35→32
11316 measured reflections	*k* = −38→38
1620 independent reflections	*l* = −8→8

### Refinement

**Table:**

Refinement on *F*^2^	Primary atom site location: structure-invariant direct methods
Least-squares matrix: full	Secondary atom site location: difference Fourier map
*R*[*F*^2^ > 2σ(*F*^2^)] = 0.039	Hydrogen site location: inferred from neighbouring sites
*wR*(*F*^2^) = 0.079	H atoms treated by a mixture of independent and constrained refinement
*S* = 0.94	*w* = 1/[σ^2^(*F*_o_^2^) + (0.0422*P*)^2^] where *P* = (*F*_o_^2^ + 2*F*_c_^2^)/3
1620 reflections	(Δ/σ)_max_ < 0.001
182 parameters	Δρ_max_ = 0.17 e Å^−^^3^
1 restraint	Δρ_min_ = −0.19 e Å^−^^3^

### Special details

**Table:**

Refinement. Due to the absence of a strong anomalous scatterer, the Flack parameter is meaningless. Thus, Friedel opposites (1312 pairs) have been merged and the item was removed from the CIF.

### Fractional atomic coordinates and isotropic or equivalent isotropic displacement parameters (Å^2^)

**Table:**

	*x*	*y*	*z*	*U*_iso_*/*U*_eq_	
O1	0.15951 (6)	0.02666 (6)	0.6362 (3)	0.0368 (5)	
O2	0.24640 (6)	0.08960 (6)	0.0779 (2)	0.0332 (4)	
N1	0.11534 (7)	0.06900 (7)	0.3925 (3)	0.0284 (5)	
N2	0.20212 (7)	0.05684 (7)	0.3521 (3)	0.0267 (5)	
N3	0.15107 (7)	0.12779 (6)	−0.0955 (3)	0.0246 (4)	
N4	0.06933 (8)	0.10885 (8)	0.1366 (4)	0.0347 (5)	
H71	0.0405 (11)	0.0991 (9)	0.207 (5)	0.056 (9)*	
H72	0.0709 (8)	0.1246 (8)	0.023 (4)	0.021 (7)*	
N5	0.21612 (7)	0.17411 (7)	−0.5455 (3)	0.0290 (5)	
C1	0.15777 (9)	0.10106 (8)	0.0864 (4)	0.0247 (5)	
C2	0.11379 (9)	0.09303 (8)	0.2032 (4)	0.0250 (5)	
C3	0.15934 (9)	0.04947 (8)	0.4708 (4)	0.0269 (6)	
C4	0.20500 (8)	0.08359 (8)	0.1627 (4)	0.0245 (5)	
C5	0.07022 (10)	0.06475 (12)	0.5270 (5)	0.0497 (8)	
H5A	0.0523	0.0943	0.5301	0.075*	
H5B	0.0801	0.0562	0.6735	0.075*	
H5C	0.0480	0.0409	0.4675	0.075*	
C6	0.24849 (9)	0.03686 (9)	0.4392 (4)	0.0360 (6)	
H6A	0.2572	0.0524	0.5741	0.054*	
H6B	0.2760	0.0409	0.3354	0.054*	
H6C	0.2433	0.0039	0.4665	0.054*	
C7	0.18654 (9)	0.13865 (8)	−0.2257 (4)	0.0274 (6)	
H7	0.2197	0.1272	−0.2021	0.033*	
C8	0.17658 (9)	0.16845 (8)	−0.4101 (3)	0.0242 (5)	
C9	0.13029 (9)	0.18992 (9)	−0.4462 (4)	0.0321 (6)	
H9	0.1032	0.1861	−0.3479	0.039*	
C10	0.12463 (10)	0.21673 (8)	−0.6268 (4)	0.0366 (6)	
H10	0.0935	0.2319	−0.6536	0.044*	
C11	0.16425 (10)	0.22156 (9)	−0.7690 (4)	0.0393 (7)	
H11	0.1607	0.2392	−0.8968	0.047*	
C12	0.20894 (10)	0.20008 (9)	−0.7199 (4)	0.0362 (7)	
H12	0.2365	0.2039	−0.8161	0.043*	

### Atomic displacement parameters (Å^2^)

**Table:**

	*U*^11^	*U*^22^	*U*^33^	*U*^12^	*U*^13^	*U*^23^
O1	0.0386 (10)	0.0401 (10)	0.0316 (10)	0.0016 (8)	0.0004 (8)	0.0116 (10)
O2	0.0224 (9)	0.0446 (11)	0.0328 (9)	0.0016 (8)	0.0037 (7)	0.0052 (9)
N1	0.0219 (11)	0.0349 (12)	0.0284 (10)	0.0010 (8)	0.0044 (9)	0.0071 (10)
N2	0.0239 (10)	0.0303 (11)	0.0261 (10)	0.0031 (8)	−0.0012 (8)	0.0042 (9)
N3	0.0214 (10)	0.0288 (11)	0.0237 (10)	−0.0008 (8)	0.0020 (9)	−0.0003 (10)
N4	0.0223 (12)	0.0480 (14)	0.0340 (12)	−0.0001 (10)	0.0035 (10)	0.0164 (12)
N5	0.0261 (11)	0.0345 (12)	0.0263 (10)	−0.0002 (9)	0.0031 (8)	0.0030 (10)
C1	0.0237 (12)	0.0276 (13)	0.0229 (11)	−0.0003 (9)	0.0002 (10)	−0.0009 (11)
C2	0.0239 (13)	0.0261 (12)	0.0251 (12)	0.0020 (9)	−0.0016 (10)	0.0002 (11)
C3	0.0253 (14)	0.0273 (13)	0.0281 (13)	−0.0017 (10)	0.0004 (10)	0.0013 (12)
C4	0.0239 (13)	0.0249 (12)	0.0248 (12)	0.0003 (9)	−0.0003 (10)	−0.0029 (11)
C5	0.0290 (15)	0.078 (2)	0.0423 (16)	0.0069 (15)	0.0112 (13)	0.0267 (16)
C6	0.0270 (13)	0.0416 (15)	0.0393 (15)	0.0055 (11)	−0.0034 (11)	0.0085 (13)
C7	0.0237 (13)	0.0321 (14)	0.0264 (12)	0.0014 (10)	0.0002 (11)	0.0000 (11)
C8	0.0224 (11)	0.0272 (13)	0.0230 (11)	−0.0021 (10)	0.0004 (10)	−0.0026 (10)
C9	0.0264 (14)	0.0350 (14)	0.0349 (14)	0.0036 (11)	0.0006 (11)	−0.0011 (12)
C10	0.0311 (14)	0.0306 (14)	0.0480 (17)	0.0049 (11)	−0.0141 (13)	−0.0018 (14)
C11	0.0456 (18)	0.0349 (14)	0.0373 (15)	−0.0021 (12)	−0.0090 (13)	0.0100 (13)
C12	0.0395 (16)	0.0392 (16)	0.0298 (14)	−0.0032 (13)	0.0050 (12)	0.0073 (12)

### Geometric parameters (Å, °)

**Table:**

O1—C3	1.223 (3)	C5—H5A	0.9800
O2—C4	1.230 (3)	C5—H5B	0.9800
N1—C2	1.368 (3)	C5—H5C	0.9800
N1—C3	1.385 (3)	C6—H6A	0.9800
N1—C5	1.465 (3)	C6—H6B	0.9800
N2—C3	1.370 (3)	C6—H6C	0.9800
N2—C4	1.413 (3)	C7—C8	1.460 (3)
N2—C6	1.463 (3)	C7—H7	0.9500
N3—C7	1.280 (3)	C8—C9	1.394 (3)
N3—C1	1.383 (3)	C9—C10	1.374 (4)
N4—C2	1.330 (3)	C9—H9	0.9500
N4—H71	0.93 (3)	C10—C11	1.380 (4)
N4—H72	0.84 (2)	C10—H10	0.9500
N5—C12	1.334 (3)	C11—C12	1.373 (4)
N5—C8	1.354 (3)	C11—H11	0.9500
C1—C2	1.393 (3)	C12—H12	0.9500
C1—C4	1.431 (3)		
			
C2—N1—C3	122.4 (2)	H5A—C5—H5C	109.5
C2—N1—C5	120.6 (2)	H5B—C5—H5C	109.5
C3—N1—C5	116.9 (2)	N2—C6—H6A	109.5
C3—N2—C4	125.46 (19)	N2—C6—H6B	109.5
C3—N2—C6	115.73 (19)	H6A—C6—H6B	109.5
C4—N2—C6	118.74 (19)	N2—C6—H6C	109.5
C7—N3—C1	124.1 (2)	H6A—C6—H6C	109.5
C2—N4—H71	118.6 (18)	H6B—C6—H6C	109.5
C2—N4—H72	113.8 (16)	N3—C7—C8	120.6 (2)
H71—N4—H72	127 (2)	N3—C7—H7	119.7
C12—N5—C8	117.6 (2)	C8—C7—H7	119.7
N3—C1—C2	114.4 (2)	N5—C8—C9	121.8 (2)
N3—C1—C4	125.6 (2)	N5—C8—C7	114.8 (2)
C2—C1—C4	120.0 (2)	C9—C8—C7	123.4 (2)
N4—C2—N1	118.0 (2)	C10—C9—C8	118.7 (2)
N4—C2—C1	121.4 (2)	C10—C9—H9	120.6
N1—C2—C1	120.6 (2)	C8—C9—H9	120.6
O1—C3—N2	122.3 (2)	C9—C10—C11	119.9 (2)
O1—C3—N1	121.4 (2)	C9—C10—H10	120.1
N2—C3—N1	116.3 (2)	C11—C10—H10	120.1
O2—C4—N2	118.9 (2)	C12—C11—C10	117.9 (3)
O2—C4—C1	126.0 (2)	C12—C11—H11	121.0
N2—C4—C1	115.1 (2)	C10—C11—H11	121.0
N1—C5—H5A	109.5	N5—C12—C11	124.1 (2)
N1—C5—H5B	109.5	N5—C12—H12	118.0
H5A—C5—H5B	109.5	C11—C12—H12	118.0
N1—C5—H5C	109.5		
			
C7—N3—C1—C2	−179.2 (2)	C6—N2—C4—O2	−0.6 (3)
C7—N3—C1—C4	3.4 (4)	C3—N2—C4—C1	−4.6 (3)
C3—N1—C2—N4	177.2 (2)	C6—N2—C4—C1	178.8 (2)
C5—N1—C2—N4	−5.7 (3)	N3—C1—C4—O2	−0.3 (4)
C3—N1—C2—C1	−3.6 (3)	C2—C1—C4—O2	−177.6 (2)
C5—N1—C2—C1	173.4 (2)	N3—C1—C4—N2	−179.7 (2)
N3—C1—C2—N4	2.3 (3)	C2—C1—C4—N2	3.0 (3)
C4—C1—C2—N4	179.9 (2)	C1—N3—C7—C8	−177.8 (2)
N3—C1—C2—N1	−176.8 (2)	C12—N5—C8—C9	−2.0 (3)
C4—C1—C2—N1	0.8 (3)	C12—N5—C8—C7	178.4 (2)
C4—N2—C3—O1	−178.5 (2)	N3—C7—C8—N5	−174.6 (2)
C6—N2—C3—O1	−1.8 (3)	N3—C7—C8—C9	5.8 (3)
C4—N2—C3—N1	2.1 (3)	N5—C8—C9—C10	1.4 (4)
C6—N2—C3—N1	178.7 (2)	C7—C8—C9—C10	−179.0 (2)
C2—N1—C3—O1	−177.2 (2)	C8—C9—C10—C11	0.6 (4)
C5—N1—C3—O1	5.6 (4)	C9—C10—C11—C12	−1.9 (4)
C2—N1—C3—N2	2.2 (3)	C8—N5—C12—C11	0.6 (4)
C5—N1—C3—N2	−174.9 (2)	C10—C11—C12—N5	1.4 (4)
C3—N2—C4—O2	176.0 (2)		

### Hydrogen-bond geometry (Å, °)

**Table:**

*D*—H···*A*	*D*—H	H···*A*	*D*···*A*	*D*—H···*A*
N4—H71···N5^i^	0.93 (3)	2.08 (3)	2.928 (3)	151 (3)
N4—H72···N3	0.84 (2)	2.25 (2)	2.661 (3)	110.2 (18)
N4—H72···O2^ii^	0.84 (2)	2.54 (2)	3.108 (3)	125.8 (19)
C7—H7···O2	0.95	2.17	2.847 (3)	127.
C11—H11···O1^iii^	0.95	2.54	3.313 (3)	138.

Symmetry codes: (i) *x*−1/4, −*y*+1/4, *z*+3/4; (ii) *x*−1/4, −*y*+1/4, *z*−1/4; (iii) −*x*+1/4, *y*+1/4, *z*−7/4.
